# Communication in the neonatal ICU for Spanish speaking parents: a qualitative interview study

**DOI:** 10.1186/s12887-023-04301-w

**Published:** 2023-09-22

**Authors:** Emily Batton, Samantha Hurst, Carlos Ramos, Leslie Catalan, Michele Freeman, Krishelle Marc-Aurele

**Affiliations:** 1 Division of Neonatology, Department of Pediatrics, Loma Linda University, 11175 Campus St Coleman Pavilion Rm 11121, Loma Linda, CA 92354 USA; 2 Herbert Wertheim School of Public Health and Human Longevity Science, University of California, San Diego, USA; 3 Division of Neonatology, Department of Pediatrics, University of California, San Diego, USA

**Keywords:** Neonatal ICU, Limited English Proficiency, Health disparities, Interpreter

## Abstract

**Background:**

In the neonatal intensive care unit (NICU), health outcome disparities exist between patients with limited English proficiency (LEP) and those proficient in English. Our objective was to investigate the communication experience of parents with LEP in the NICU to learn how to mitigate such health disparities.

**Methods:**

A certified bilingual provider conducted seventeen interviews of parents who identified Spanish as their preferred language and whose newborn was admitted to the NICU for ≥ 1 week. Interviews were conducted August 2020 – December 2021. Conventional content analysis utilizing an inductive open coding process was performed.

**Results:**

The experiences of Spanish speaking parents with LEP in the NICU can be characterized by 3 main themes: 1) Information accessibility 2) Perspectives about interpreters and 3) Emotional consequences.

**Conclusions:**

Our findings can inform neonatal quality initiatives to facilitate timely and good communication for NICU families with LEP.

## Background

Effective and timely communication is required for optimal medical care and is a right afforded by the United States’ legal system to all patients regardless of their preferred language [1, 2]. Unfortunately, many patients with limited English proficiency (LEP) receive inappropriate interpretive services, resulting in confusion regarding diagnosis and treatment, and negative experiences with the healthcare system [3, 4].

Discordant language preferences between providers and parents affect families of pediatric patients, including those in the Neonatal Intensive Care Unit (NICU). Spanish speaking parents with LEP are 4 times more likely to misunderstand their newborn’s diagnosis and are less emotionally and technically prepared for NICU discharge [5–7]. Data on this topic is sparse in the neonatal literature and there is a need to better understand the specifics of how LEP contributes to health outcome disparities. Identifying the mechanisms of these disparities and implementing solutions are important in achieving optimal health outcomes of patients discharged from the NICU, many of whom experience prolonged hospitalizations, have medically complex health problems and require long term follow up with several subspecialists.

Our objective was to investigate the communication experience of parents with LEP in the NICU. The primary aims were to 1) explore how Spanish speaking parents with LEP receive information about their baby 2) identify what information they receive and how this impacts participation in their baby’s care and 3) assess satisfaction with communication and how communication can improve when the preferred languages of the parent and provider differ.

## Methods

### Study design

We conducted a qualitative study with the goal of exploring and characterizing parent-lived experiences. In-person interviews were performed at a single-center, level III, 52-bed NICU in San Diego, CA where in-person interpreters are not readily available and providers often communicate with families by telehealth interpreter modalities. Enrollment started in August 2020, six months after the declaration of the Coronavirus Pandemic.

### Sampling and eligibility

**Fig. 1:**
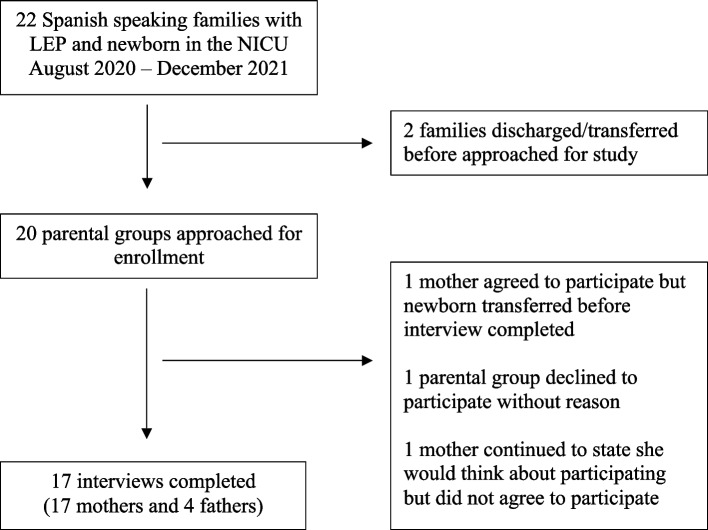
Represents the enrollment process conducted during this study including those eligible for enrollment and reasons that eligible individuals were not enrolled in the study

We performed criterion purposeful sampling for this study. Parents were eligible for enrollment if their newborn was admitted to the NICU for at least 1 week and if they identified Spanish as their preferred language without being English proficient. Language preference was determined by the admitting bedside registered nurse and later confirmed by a study investigator at time of enrollment. Parents who preferred a language other than Spanish were excluded to limit the number of interviewers needed and to standardize the interview experience. If one parent was bilingual and another had LEP, parents were permitted to be interviewed together to allow for emotional support. Enrollment was limited to parents of newborns admitted for at least 7 days to allow for sufficient interactions between parents and the medical team. Attempts were made to enroll all eligible families during the study period. Due to scheduling difficulties, we were unable to approach 2 eligible families for enrollment prior to transfer of their newborns to other facilities. Non-participation is discussed in Fig. 1. Informed consent for study participation was obtained using trained Spanish medical interpreters. IRB approval was obtained.

### Interviews

Interviews were conducted using a semi-structured interview format to guide discussions (Table 1). All interview questions were derived from the specific aims of the study. Five pilot interviews were conducted to assess the interview guide for language clarity, question relevance, and redundancy. Revisions were made accordingly. Interviews were scheduled at a time per parent’s preference and were conducted by one of two certified bilingual providers (a NICU respiratory therapist and a NICU social worker). Parents were offered the opportunity to complete the interview at their child’s bedside or in a private room. All interviews were audio-recorded (Sony ICDUX560 Digital Voice Recorder) for analysis.

**Table 1:** Semi-Structured Interview Guide (Final Version)

Interview Question	Corresponding Aim
So far, how often have you received information about your baby? Prompt: every day, 3 times a week, once a week etc Has it been from the doctor or the nurse? How often do you wish you received information about your baby? How many times have you talked to the doctor about your baby? If the nurse does not speak Spanish, how do you communicate with her or him? How do you let them know you have questions? Can you give me some examples? If you call the NICU and no one speaks Spanish, how do you communicate with the medical team? Tell me about your experience using the interpreters in the NICU Prompts: Do you feel well understood? Do you think the interpretation is accurate? Has it been a positive or negative experience? Has there been a situation when you did not want to use an interpreter? Prompt: Can you tell me more?	Explore how parents with LEP receive information about their baby
Tell me about your baby. How is he/she doing? What have you been told about why your baby needs to be in the hospital? What part of your baby’s care have you learned to do so far? If the nurse does not speak Spanish, how do you participate in your baby’s care? How do you think the language difference between you and the medical team affects how much you learn about your baby? Can you give examples?	Identify what information parents with LEP receive and how this impacts their participation in their child’s care
When you wanted more information about your baby, how could we have made it easier for you? Have you ever felt unable to ask all the questions you wanted and can you tell me about that experience? What could we do better to communicate with families who speak a language different from English?	Assess parent satisfaction with communication and how they feel communication could improve when the language preferences of the parent and medical team differ

We chose a NICU respiratory therapist and social worker to perform the interviews due to their availability as well as their familiarity with NICU patients and their families. We intentionally chose non-physician interviewers to minimize the potential influence of the doctor-patient hierarchy on interview responses. We are not aware of any enrolled families that were inhibited during the interview. Families that were hesitant to share their experiences did not consent to the study.

Seventeen interviews of 17 mothers and 4 fathers were performed (Fig. 1). One mother declined to participate during the early weeks of her daughter’s hospitalization, but agreed to participate closer to discharge. All interviews were performed at the newborn’s bedside at the parent’s request. The majority of the interviews were between 6 and 9 min in length [range 5–22, mode 6]. Of the mothers included, 15 (88%) spoke little to no English, one was bilingual (Spanish and English), and one preferred Spanish but often spoke to the medical team with limited English. Of the fathers included, two (50%) spoke little to no English, one was bilingual (Spanish and English), and one preferred Spanish but frequently spoke to the medical team with limited English.

Quantitative and qualitative data about the newborns of enrolled parents were collected to demographically represent the sample. Demographic and illness severity data for the corresponding infants in this study are in Table 2. There were three sets of twins and 20 infants total. Seventeen infants (85%) had a primary diagnosis of prematurity, including three infants born < 28 weeks’ gestation. One infant was admitted for neonatal encephalopathy and two for evaluation of septo-optic dysplasia. All except three infants required IV medications or nutritional support during their hospitalization; however, only two infants required IV support at the time of the interview. Thirteen (65%) of the infants required some level of respiratory support during their hospitalization and seven (35%) required intubation, but at the time of the interview, only three (15%) required non-invasive respiratory support and none were intubated. All infants survived to NICU discharge.

**Table 2:** Newborn characteristics (*n* = 20)

Birthweight: Average grams (range)	**1877 (550 – 4050)**
Birth Gestational Age: Average weeks, days (range)	33,2 (25,1 – 41,2)
Sets of multiples	3 (twins)
Female: n (%)	10 (50)
1 Minute Apgar: Median (range)	6 (1–9)
5 Minute Apgar: Median (range)	8 (5–9)
Mild SNAPPE-II Score^a^ at 12 HOL: n (%)	17 (85)
Required intubation during hospitalization: n (%)	7 (35)
Room air at time of interview: n (%)	17 (85)
Length of stay: Average days (range)	45 (9 –120)
Day of life at interview: Average days (range)	31 (3 – 118)

^a^Score for neonatal acute physiology-perinatal extension II

### Data transcription and translation

All interviews except one were transcribed verbatim into Spanish using a health insurance portability and accountability act (HIPAA) compliant professional transcription service (TranscribeMe). Consent to use TranscribeMe could not be obtained for the first interview; therefore, this interview was transcribed using Google’s HIPAA compliant speech to text program according to the original IRB protocol. Health information was removed from the Spanish transcripts and the transcripts were then reviewed for errors. Spanish transcripts were translated into English using Google’s Translate Application Programming Interface and then reviewed again for accuracy.

### Data analysis

Conventional content analysis utilizing an open coding process by an inductive approach was performed to distinguish patterns and concepts represented in the final results [8, 9]. This type of inductive analysis was considered appropriate for this study given the limited research and knowledge on this particular topic. Multiple cycles of general and focused coding were performed with a team-based approach and guided by an expert in qualitative methodology. Disagreements among coders as to the assignment or accuracy of the coding labels were resolved through discussion and consensus of the team.

## Results

Qualitative analysis identified three main themes: The experiences of Spanish speaking parents with LEP in the NICU can be characterized by 1) information accessibility 2) perspectives about interpreters and 3) emotional consequences. Theme descriptions are elaborated with supporting quotes that exemplify nuances of the participants’ lived experiences. Quotes are verbatim unless indicated by an ellipsis (…) to signal that small segments of the text have been removed for clarity.

### Information accessibility

Parents described communication with the medical team as difficult. Parents had challenges obtaining information directly from doctors and all but one family stated they primarily received information from nurses. One family was unaware that doctors were present at night and stated they had not spoken to or seen a doctor in weeks. At times, parents understood medical updates in English, but felt unable to express questions and concerns in English, which hindered their ability to connect with the medical team and left them unprepared for the next step in their child’s care. For example, one mother understood that her son required closure of his patent ductus arteriosus, but felt she could not ask questions about the procedure because of her LEP.

*“It was difficult for me because I do not know English. I cannot express myself with them as I am expressing myself with you (…) That was the only thing that made it difficult for me not to be able to talk to them.”* (Mother)

*“Maybe, in my mind there were doubts, but because I didn't know how to say them in English, I didn't ask (…) maybe if it would have been all in Spanish, I would have asked more questions. The language does stop you a little bit.”* (Mother)

Parents reported using signals with the nurse in order to communicate when they were unable to understand each other with words. They reported mimicking the nurse’s motions, learning how to care for their child without speaking to one another. Parents alluded to extensive energy required by them and the nurses to communicate, using terms such as “struggle”, “by force” or “try harder”.

*“I am the one who is the most self-conscious when I speak English. I understand you (…) but to speak it, sometimes, it is more difficult for me. But still, I just try harder to get my words out and that’s how I communicate.”* (Father)

Parents perceived delays in communication as a result of not speaking English. Parents reported waiting for the telehealth interpreter to connect to the internet, to find an ad hoc interpreter (most often a bilingual nurse) when visiting in person, and for the medical team to return their phone call with an interpreter, particularly at night. While some parents were able to call from home and request an interpreter with their limited English, others could not, meaning medical updates were delayed until parents visited in person.

*“Sometimes we have called, and since the nurse only speaks English, she tells me that she is going to return the call with an interpreter, but the call takes about half an hour or more to return (...) Or sometimes they don't call me back anymore.”* (Mother)

Ten parents directly stated they were more likely to receive information when they were present in the NICU and when they asked the medical team for an update.

*“I have received information since she was born. I always received it, every third day, that I came to the hospital.”* (Mother)

*“When I arrive, there is always a nurse here and she gives me the summary of everything, how the girls have been during the night, during the day, what has progressed, what has not. All the information is given to me as soon as I arrive.”* (Mother)

One family felt they did not receive information about their child unless they directly asked the medical team a specific question.

*“They told me to sign about the vaccines a week ago and I haven’t seen them give the vaccines. So, I have to ask ‘(…) when are they going to give it to him?’(…) Things like that, if we don't ask, they don't tell us.”* (Father)

Despite the perceived difficulty and delay in obtaining information, parents appeared well informed about their newborn’s admission diagnosis and understood their child’s clinical status. While 9 parents felt the language difference hindered their ability to learn about their baby and ask questions, almost all parents reported being extensively involved in their child’s care, performing tasks such as diaper changes, temperature checks and feeding their child. The majority of parents were satisfied with how often they spoke to the medical team and only 2 parental groups desired more frequent updates. In other words, when parents wanted information, they wished they were able to obtain it quicker; however, they reported feeling overall satisfied with how often they were updated by providers.

### Perspectives about interpreters

Parents mostly used telehealth interpreters (video and audio) and were rarely offered in-person interpreters. In-person interpreter staff at the study hospital was limited: often not available or difficult to schedule. Although parents stated that telehealth interpreters were helpful, parents feared that interpreters incorrectly conveyed parental concerns and information from the medical team.

*“I feel that sometimes the interpreters do not explain medical things well, but since I speak a little English, I do understand them, and what they are translating for me is not what the doctor is saying."* (Mother)

Only one parent declined the offer to use an interpreter; however, many parents did not request an interpreter for simple tasks because it was faster to communicate in their limited English than wait for an interpreter. Parents weighed the ability to ask questions through an interpreter with the time required to wait for an interpreter to become available. Parents desired access to more bilingual providers and written material about their baby hoping this would provide accurate communication without the need to wait for an interpreter.

*“I think that with a doctor who speaks Spanish, it is easier because it takes long to look for the person who comes to interpret for you.”*(Mother)

### Emotional consequences

Parents reported negative feelings such as shame, frustration and uncertainty associated with their LEP. They alluded to a complex emotional response including fear of judgment when admitting they did not understand what the team had said, which stopped them from asking the medical team for clarification.

*“If I do not understand a particular word, I keep it to myself as I do not know how to ask. Many times one is ashamed to say: ‘What? What did you say?’, Because you think that they will not understand you.”* (Mother)

*“Sometimes (…) there are words that I don't know how to say. Then you get frustrated, and sometimes I feel like some nurses (…) I don't know if it's because you're Hispanic, they treat you differently.”* (Mother)

*“Sometimes you think that: "if I don't know English, what are they going to think of me or what are they going to say?”* (Mother)

One mother who spoke non-fluent English and declined an interpreter at the beginning of her child’s hospitalization, experienced regret with her initial decision. She stated that as the hospitalization continued, she wished the team had been more insistent on using an interpreter from the beginning instead of placing the responsibility on her to admit she needed an interpreter.

*“Perhaps, at the beginning of everything, you [medical team members] should ask several times: ‘Are you sure that you understand?’ or ‘We can really bring an interpreter.’ Maybe you should insist a little more on bringing the interpreter (…) because sometimes you need a little push to accept it.”* (Mother)

Parents reported relying on family members to communicate with the medical team, placing the burden of interpreting medically complex information on untrained individuals. Several parents relied on their spouse to interpret for them, which left them unable to communicate with the team when their spouse was not present. One mother reported relying on her daughter to help her communicate by phone when not present in person, stating that if her daughter was not available to help interpret, she would wait to receive information in person.

## Discussion

This study provides insight into an experience that is poorly understood and rarely investigated in the medical literature. To our knowledge, this is the first published study that focuses solely on the communication experiences of Spanish speaking NICU parents with LEP in a qualitative interview manner. Findings from this study can identify strategies to provide more equitable care to NICU families with LEP. Recommendations for improvement are summarized in Table 3. Although recommendations are displayed in relation to individual themes, we recognize that there is significant overlap of our themes and their solutions.

**Table 3:** Study findings and recommendations

Study Finding	Related Theme	Recommendation
Parents received information primarily from nurses	Information Accessibility	Improving parent to nurse communication should be prioritized when parents have LEP
LEP hindered parental abilities to learn about their baby, ask questions and express concerns, particularly when updates were provided in English	Information Accessibility	Regularly scheduled updates with an interpreter should occur. Parents should be provided a way to inform staff that they have questions, even when an interpreter is not present
Parents had difficulty obtaining information remotely and were more likely to receive information when present in the NICU	Information Accessibility	Units should implement a way parents can remotely request updates from the medical team with an interpreter at all hours
Parents desired access to more bilingual providers to avoid delays in communication and miscommunication related to interpreter use	Perspectives About Interpreters	Hiring and training bilingual providers should be prioritized. If bilingual providers are not available, the use of ad hoc interpreters should be discouraged. The teach back method should be used to ensure adequate communication with interpreter use
Parents experienced negative emotional consequences related to their LEP	Emotional Consequences	Medical teams should default to using the parent’s preferred language regardless of perceived English fluency. Units should standardize how parental preferred language is determined. The need for an interpreter should be reevaluated throughout a hospital stay if the use of an interpreter is initially declined by the parent

Our study showed that parents primarily received information from nurses, a finding that appears true regardless of the parents’ preferred language. Studies show that nurses are more available, information from doctors declines over time during a hospital stay, and that parents often ask nurses for clarification after speaking with the doctor [7, 10]. These findings suggest that improvement initiatives in the NICU should focus on the nurse-to-parent relationship in order to best optimize communication between the medical team and families with LEP.

Over half of the parents in our study felt the language difference limited how much they learned about their baby. Parents also felt their LEP hindered their ability to ask questions and express concerns. This was particularly true when updates were provided in English to Spanish speaking parents with LEP, which unfortunately has been shown to occur more than 50% the time [5]. Our findings are consistent with prior literature [7, 11–13]. A survey by Khan et al. found that families with LEP are 2 times more likely to be afraid of asking questions when “something does not seem right”, 4 times less likely to “freely speak up” if they see something that may negatively affect care, and 5 times less likely to “question the decisions or actions of healthcare providers” [14].

Based on our results, we recommend implementing a structured system for parents to receive regularly scheduled updates in their preferred language. This may be during routine rounds or separate meetings with an interpreter scheduled on the same day each week. Physicians should lead these scheduled updates to ensure frequent communication between parent and physician. Nursing staff should also have time to connect with families through an interpreter on a daily basis. This may require a different staffing model (i.e., 2:1 patient-to-nurse ratio, or dedicated communication resource nurse) in order to communicate with families with LEP. Parents should also be provided a way to easily notify the team at bedside that an interpreter is needed because they have questions or concerns. This may facilitate parental involvement and empower them to ask questions about their baby’s care.

Our study adds to the literature by demonstrating that parents with LEP had difficulty obtaining information remotely, particularly at night. Our study also showed that parents with LEP were more likely to receive information while present in the NICU and after prompting the medical team for an update. Many parents in the NICU desire routine, timely and clear information about their child’s condition [10, 15], but this cannot be achieved if parents are required to be in person to receive information. NICU hospitalizations often span months at a time and parents frequently need to return to work or care for other family members away from the hospital, preventing them from being at the bedside. Our study highlights the need to facilitate timely updates for parents with LEP when they are away from the hospital.

We strongly encourage each unit to implement a way families can reach the medical team from home with an interpreter to avoid waiting for a call back or waiting until they can visit and obtain information in person. This may be as simple as routinely providing the phone number for a hospital’s telephone interpreter service. Certain electronic medical records may also offer a way parents can send messages to the medical team from home in their preferred language.

Parents in our study reported a strong desire to have more bilingual providers available to avoid delays in communication and miscommunication associated with interpreter use. Evidence shows that patients seen by bilingual physicians are more likely to ask questions and have improved patient recall when compared to patients who utilize a medical interpreter [16]. More bilingual providers may also facilitate casual conversations that have been shown to provide English speaking parents with emotional relief [10] and foster trust with the medical team. We therefore recommend prioritizing the training and hiring of bilingual providers to facilitate communication for parents with LEP. To ensure accurate communication occurs when an interpreter is used, we recommend utilizing the ‘teach back method’ to clarify what information the interpreter conveyed. This can be done by asking the family to explain what they understand about the information provided to them. We also discourage the use of ad hoc or untrained interpreters whenever possible because the use of such individuals has been shown to be detrimental to health outcomes [17–21].

Finally, our study emphasized the negative emotional consequences such as shame, frustration and fear of judgment that parents experienced as a result of their LEP, findings that are consistent with prior literature [11, 22]. These feelings can affect parental confidence in a child’s physician, negatively impact health care outcomes [23], and due to a lack of communication, can make parents feel excluded and deprived of the role of being a primary caregiver [10, 24]. Therefore, providers must be sensitive to the stress that LEP can add to an already emotionally difficult time in a parent’s life.

To avoid such negative consequences, we recommend routinely defaulting to the parents’ preferred language. This should be done despite any perceived English fluency because perceptions of parental English fluency are often incorrect [25]. If a parent initially declines an interpreter, reevaluating the need for an interpreter throughout a hospitalization is important. We also recommend each NICU evaluate their method of determining parental language preference upon admission, ensuring that the process is completed in an objective, standard, and judgment-free manner that allows parents to answer honestly.

There are several strengths to our study. Our study’s narrow aims and specific target population allowed data sufficiency to be reached with a smaller number of participants [26]. We standardized the interview process by limiting the number of interviewers to two individuals and conducted the interviews at the parents’ convenience to maximize their comfort and allow for honest and complete responses to our questions.

There are several limitations to our study. Occasional recorder malfunction resulted in a loss of minor information from our interviews. The COVID-19 pandemic visiting restrictions may have limited enrollment. Our study had definite logistical boundaries with the inability to extend the enrollment period beyond December 2021 because study completion was needed for trainee graduation. Difficulty coordinating schedules to perform interviews resulted in a few missed opportunities in enrollment. Finally, interviewers were not formerly trained in qualitative interviewing, which may have resulted in shorter interview length. Although short interview length could raise concerns regarding the rigor of our study and whether parents felt inhibited from answering questions honestly, the interviewers of this study were specifically discouraged from coercing parents into sharing more information. While this is a limitation of our data, it was done in an attempt to empathize with the positionality of the parents.

## Conclusion

This is a single center, qualitative study that utilized in-person interviews to investigate the communication experience of Spanish speaking parents with LEP, providing insight into unique challenges that such families face in the NICU. Our findings can inform neonatal quality initiatives to decrease delays and burdens for families with LEP to receive timely, accessible, and good communication. Future studies can use the information we learned to determine the best strategies to facilitate communication with Spanish speaking families.

## Data Availability

The datasets used and/or analyzed during the current study are available from the corresponding author on reasonable request.

## Abbreviations

LEP: Limited English proficiency
NICU: Neonatal intensive care unit
HIPAA: Health insurance portability and accountability act
